# A Rare Case of Thyroidea Ima Arising From the Internal Thoracic Artery and Reaching Up to the Parathyroid Gland

**DOI:** 10.7759/cureus.49551

**Published:** 2023-11-28

**Authors:** Lyubomir Gaydarski, Mihail Angelov, Yoanna Tivcheva, Nikolay Krastev, Boycho Landzhov

**Affiliations:** 1 Department of Anatomy, Histology, and Embryology, Medical University of Sofia, Sofia, BGR

**Keywords:** thyroidea ima artery, parathyroid blood supply, neck endocrine surgery, anatomical variations, vascular variations

## Abstract

The thyroidea ima artery (TIA), also known as the Neubauer artery, is a variable artery that takes part in the blood supply of the thyroid gland. The overall prevalence rate of the thyroidea ima artery is 3.3%. Though it most commonly branches off the brachiocephalic trunk or the arch of the aorta, the artery has a highly variable origin point. Herein, we present a rare case of a thyroidea ima originating from the left internal thoracic artery, found during routine dissection of a Caucasian, 76-year-old, male, formalin-fixed cadaver. The artery is a normal finding during embryological development as a part of a more extensive network of vessels supplying the thyroid gland. The latter undergoes significant remodeling, and only four superior and inferior thyroid arteries remain. Thus, the presence of the thyroidea ima is considered to be due to changes in the said remodeling process. Due to its course, anteriorly to the trachea, the thyroidea ima artery might pose problems during different surgical procedures regarding the inner anterior region of the neck, such as tracheotomy, thyroidectomy, laryngeal transplantation, and selective embolization of the thyroid arteries (SETA). Furthermore, complications during the operative treatment of parathyroid conditions and mediastinal bleeding are to be expected. The variable presence and origin of the thyroidea ima have clear and significant clinical and surgical implications. They must be considered when procedures in the neck's anterior region occur.

## Introduction

The thyroid gland is an unpaired endocrine gland with a rich vascular network [1]. The typical blood supply to the thyroid gland involves the superior thyroid artery (STA) and inferior thyroid artery (ITA) [2]. However, in uncommon instances, an alternative blood supply to the anterior part of the thyroid gland is provided by the thyroidea ima artery (TIA). Professor Johann Ernst Neubauer initially described the TIA in 1772, leading to its alternative designation as the Neubauer artery [3]. This particular artery is observed with a mean incidence rate of 3.3% in the general adult population. It predominantly originates from the brachiocephalic trunk, accounting for 74% of cases, followed by the right common carotid artery (CCA) (7.7%), the aortic arch (4.8%), the left common carotid artery (1.9%), and the left internal thoracic artery (1.9%). An exciting trend noted in the research is that TIA is more likely to originate from arteries on the right side (88.5%) [3]. Our article features a rare case of TIA's origin from the left internal thoracic artery. In order to better comprehend the sophisticated relations between the different variants in the origin of TIA, we need first to understand the complex embryological development of the thyroid gland.

The thyroid gland undergoes embryological development between the fourth and seventh weeks of gestational development. It starts as a midline thickening of the floor of the fetal pharynx. During the next three weeks of development, it progressively descends until it reaches the lower neck between the second and fourth tracheal cartilages. Along its pathway, it stays connected to its original point of origin through the thyroglossal duct. During this time frame, an extensive network of vessels is present, all of which disappear after the end of the development of the fetal gland except for the standard STA and ITA [4]. The TIA is not an uncommon finding within this extensive network of vessels. However, it usually disappears after the four main arteries are shaped [5]. TIA is important from an anatomical point of view since its morphological characteristics can be directly correlated to the distribution of the vessels within the gland itself [6]. A present TIA can alter the morphology of the gland, which must be kept in mind from a clinical point of view when assessing the characteristics of the gland through ultrasound or any other imaging modalities, as well as from an anatomical point of view. This correlation between vascularization and morphology also has implications from a biotechnological point of view when attempting to create a bioartificial thyroid gland [7]. The parathyroid glands (PTG) consist of four small, flattened structures, each measuring a few millimeters in diameter [1]. They are positioned on the posterior surface of the thyroid gland. Generally, the ITAs supply blood to the parathyroid glands [8]. However, there are few cases of TIA supplying the parathyroid gland [9,10]. Moreover, TIA has the utmost clinical significance. The presence of TIA, due to its unique origin and path, can lead to significant complications in standard surgical procedures such as tracheotomy [11]. Therefore, a comprehensive understanding of this artery is crucial for medical practitioners performing procedures in the anterior neck region.

## Case presentation

**Figure 1:**
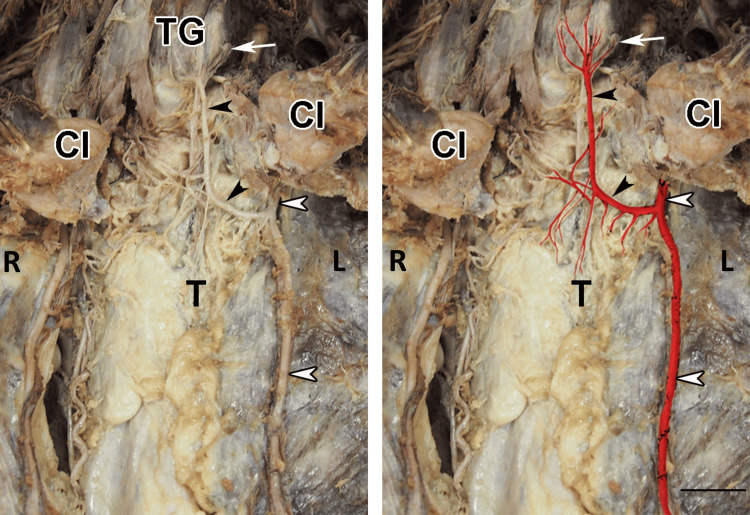
Thyroidea ima artery (TIA) (black arrowheads) originating from the internal thoracic artery (white arrowheads). The following branches of TIA are visible: superior thymic branches entering the thymus (T), muscular branches for the adjacent infrahyoid muscles, thyroid branches for the thyroid gland (TG), and parathyroid branch ascending to the left parathyroid gland (white arrow). Scale bar: 2 cm. Cl, clavicle; R, right; L, left

While conducting a routine dissection of a formalin-fixed cadaver of a 76-year-old Caucasian male at the Department of Anatomy, Histology, and Embryology at the Medical University of Sofia, we observed an aberrant vessel extending toward the thyroid and parathyroid glands. Its characteristics align with the TIA descriptions documented in the medical literature. After a detailed dissection and the removal of connective and fat tissues surrounding the artery, it was determined that this particular TIA originates from the left internal thoracic artery. The variable artery follows an initial transverse course, then curves, and finally takes an ascending and medial path. It passes anteriorly over the trachea, branching off into a network of vessels at the inferolateral border of the left lobe of the thyroid gland, thereby providing blood supply to this specific area (Figure 1).

Measurements were taken using a standard ruler to measure the length of the variant artery. The total length of the artery is 6.7 cm, comprising a transverse part of 1.9 cm and an ascending part of 4.8 cm. The artery's lumen measures 3.6 mm. Additionally, the TIA gives rise to the following branches: superior thymic branches (entering the thymus), muscular branches (for the adjacent infrahyoid muscles), thyroid branches for the inferior part of the thyroid gland, and one small branch reaching to the left parathyroid gland, parathyroid branch. Upon completing a meticulous dissection of the anterior neck region and upper thorax, we determined that both STAs and ITAs were present, with no variations in their origin sites. There was no available medical or surgical background information for the cadaver, and no visible scars were noted before the dissection.

## Discussion

The current article features a rare case of TIA originating from the left internal thoracic artery, which ascends and provides a solitary branch for the left inferior parathyroid gland. Although the usual blood supply for the PTG comes from the STA and ITA branches, there are rare instances of the TIA providing branches for the PTG. Krudy et al. documented three radiological cases where the TIA supplied branches for the inferior PTG [10]. In two instances, the anomalous TIA originated from the brachiocephalic trunk, and in the third case, it arose from the right common carotid artery. In the first case, Krudy et al. highlighted that the left inferior PTG received blood supply solely from a branch of the TIA originating from the brachiocephalic trunk. They emphasized the crucial role of TIA in supplying blood to PTG tumors, stressing the importance of recognizing the possibility of TIA presence. While the selective embolization of vessels supplying blood to PTG tumors is an effective treatment, it may be compromised if branches of an aberrant TIA are not identified and embolized [10]. Given that the typical blood supply for the PTG involves solitary arteries originating from the ITA [8], the presence of a TIA, especially in conjunction with a hypoplastic ITA, as reported by Krudy et al., would make the TIA the main feeding artery for the inferior PTG [10]. In such scenarios, the presence of a TIA becomes crucial in predicting outcomes after total thyroidectomies, specifically anticipating the risk of transient or permanent hypoparathyroidism.

Furthermore, if hypoplastic or missing ITAs accompany a TIA, any damage to the TIA would result in the devascularization of areas solely supplied by the TIA. Employing pre- or intraoperative angiography [12] to assess the vascularization of both the thyroid and parathyroid, including any anatomical variations such as TIA, is essential for achieving positive postoperative outcomes and avoiding complications. Notably, Totlis et al. recently reported a case of TIA originating from the right common carotid artery (CCA), providing branches for the inferior pair of PTG [11]. Recently, Novakov and Delchev reported an interesting case of large TIA originating from the brachiocephalic trunk and replacing the bilaterally absent ITAs [13]. However, our case presents a distinctive situation, with TIA arising from the left internal thoracic artery, providing a sole branch for the left inferior PTG. This specific case has not been reported previously.

TIA is a highly variable vessel. A comprehensive meta-analysis incorporating 36 studies involving 4335 subjects, conducted by Yurasakpong et al., calculated that the prevalence of TIA in adults is approximately 3.3% [3]. In the study of Yurasakpong et al., the incidence rate of TIA originating from the left internal thoracic artery was calculated to be 1.9% of all accounts of present TIA [3]. On the other hand, Natsis et al., in a systematic review, determined that TIA's prevalence was 2% [14]. The same study observed that the TIA could also have its origin in the pericardiophrenic artery, the subclavian artery, the thyrocervical trunk, the inferior thyroid, or the transverse scapular artery [14]. A large-scale study by Toni et al. found that certain ethnic groups exhibit a higher incidence of TIA and other thyroid vascular anomalies. Specifically, Asians demonstrated a higher incidence rate than Caucasians (10% versus 6%, p < 0.05). Regardless of the ethnic group, the brachiocephalic trunk remained the most frequent origin point for TIA in both populations [15].

The existence of the TIA poses a risk for significant complications in median surgical procedures, including tracheotomy, thyroidectomy, and laryngeal transplantation [6,15]. The classical approach for partial or total thyroidectomy involves a horizontal collar incision proximal to the upper border of the sternum, going through the subcutaneous tissue, cutting the platysma, and dividing the infrahyoid muscles vertically in order to reach the thyroid gland [16]. In recent years, minimally invasive approaches have been developed. Such approaches involve the usage of endoscopes inserted through small incisions either in the axillary fossa, in the chest, in the areola, or even through the oral cavity floor [16]. Nevertheless, a present TIA might pose serious problems if not correctly identified during all the described surgical approaches.

Several complications can manifest as challenging-to-control bleeding, leading to severe consequences such as mediastinal hemorrhages. Notably, the risks are heightened when the TIA originates from the aortic arch due to the vessel's high-pressure nature. The cut vessel tends to retract below the manubrium of the sternum, exacerbating the severity of bleeding in such cases. Furthermore, even if the aberrant TIA is not damaged during the surgical approach (in the case of the transoral approach), if the thyroid branches of TIA are not correctly ligated before the total thyroidectomy, severe bleeding might occur [16]. Moreover, TIA also has an important role when trying to control bleeding from chest and neck trauma. The evaluation and control of mediastinal bleeding with traumatic origin should include a thorough evaluation of the TIA. Should any injury to the TIA be present, the treating physician should consider performing an embolization of the injured artery to reduce or outright stop the bleeding into the mediastinum [17]. A thorough understanding of the presence and origin point of the TIA is crucial for medical procedures focused on thyroid tissues, such as selective embolization of the thyroid arteries (SETA). SETA is employed as a palliative treatment for inoperable anaplastic thyroid carcinoma [18]. This detailed knowledge is essential for ensuring the precision and effectiveness of such therapeutic interventions. Furthermore, identifying the arteries supplying the parathyroid gland (PTG) is vital to prevent postoperative complications, notably hypocalcemia resulting from parathyroid gland devascularization after thyroidectomy. This surgery commonly leads to transient or permanent hypoparathyroidism [19,20].

## Conclusions

In summary, our study sheds light on a rare case of the TIA originating from the left internal thoracic artery in a 76-year-old cadaver. Our study emphasizes the clinical implications of such variations, especially in surgical procedures in the neck's anterior region. The TIA's potential to supply the parathyroid gland has significant consequences for thyroidectomies. Therefore, pre- or intraoperative angiography is of utmost importance for successful outcomes. The detailed analysis contributes valuable insights into the anatomical variations of TIA, enhancing understanding for medical practitioners and aiding in the precision of surgical interventions, particularly in the context of thyroid and parathyroid procedures. Overall, our study contributes valuable insights into the anatomical nuances and surgical implications of the TIA, urging careful consideration in clinical practice.
